# Retroviruses, retroelements and their restrictions

**DOI:** 10.3389/fmicb.2013.00197

**Published:** 2013-07-15

**Authors:** Atsushi Koito, Yukihito Ishizaka

**Affiliations:** ^1^Department of Retrovirology and Self-Defense, Kumamoto UniversityKumamoto, Japan; ^2^Department of Infectious Diseases, National Center for Global Health and MedicineTokyo, Japan

HIV and HTLV have been recognized as important pathogens because of their association with lethal diseases in human: HIV causes Acquired Immunodeficiency Syndrome (AIDS) and HTLV is the etiological agent for adult T-cell leukemia. Considerable resources and efforts therefore have been directed at understanding the interaction between these human retroviruses and their host which may provide clues as to how the infection can be controlled or prevented. Among the key scientific successes is the identification of intracellular “restriction factors” that have evolved as obstacles to the replication of pathogens including infectious retroviruses. The discovery of APOBEC3 cytidine deaminases, which are strong mutagens of retroviral genomes and intracellular retroelements, opened a new era of intense research activities into the spectrum of intrinsic anti-HIV activity, leading to the identification of TRIM5α, BST2/Tetherin, and SAMHD1. In response, HIV has evolved several accessory genes as weaponries to evade or antagonize these intracellular restriction activities. This issue of Research Topic covers several reviews of the mechanisms of these restriction factors and their counter-balance by HIV-1 gene products. The anti-HIV-1 control mechanism by APOBEC3 cytidine deaminase is explained in the paper by Imahashi et al. (2012), while the article by Takaori-Kondo and Shindo (2013) discusses the functions of the viral infectivity factor (Vif) in the HIV life cycle and its integral role in antagonizing APOBEC3. In Sato et al. (2012) review, the interaction between HIV-1 Vpu and other viral proteins with BST2/Tetherin is presented, and Fujita et al. (2012) describe the functions of HIV-2/SIV encoded Vpx and how it counteracts SAMHD1 to facilitate viral replication. TRIM5α - and Fv1-mediated restriction activity that targets the incoming viral capsid to prevent uncoating is reviewed by Nakayama and Shioda (2012), and Suzuki et al. (2012) discuss host factors that restrict retroviral integration. In addition, investigations into the export of HIV and HTLV genomic RNA from the nucleus are summarized by Shida (2012), and animal models on HTLV and related retroviruses are evaluated by Hajj et al. (2012).

The intracellular antiretroviral defenses evolved in response to endogenous retroelements that make up more than 40% of the entire mammalian genome, and which are regarded as ancestors of infectious retroviruses. Long term repeat (LTR)-type retroelements are present in all higher eukaryotes, representing about 8% of the human genome. The critical role played by LTR retrotransponsons in “hijacking” retroviral genes to shape mammalian evolution is described by Kaneko-Ishino and Ishino (2012). Non-LTR retroelements can be found at extremely high copy numbers also, with a significant portion of mammalian genomes consisting of long interspersed elements (LINEs). Mammalian genomes are modified by LINEs through insertions, but also by the indirect replication of non-autonomous retrotransposons such as short interspersed elements (SINEs). LINEs insertion was shown to have played, and continue to play important roles in genomic evolution and somatic genome mosaicism-mediated physiology such as brain activity in mammalian species. Despite the impact of LINEs insertion, much of the process of LINEs rertrotransposition remains unexplored. The contribution by Ishizaka et al. (2012) describes the cellular factors required for the LINEs rertrotransposition. And, because retrotransposition can confer genetic diversity that is beneficial to the host, the vertebrate intrinsic immunity has evolved to support a balance between retroelement insertions that confer beneficial and those that cause deleterious gene disruptions. Arias et al. (2012) summarize our current understanding of the anti-retroelement and antiretroviral activities and functions of human APOBEC3s. The mechanism of action of not only APOBEC3 but also of other APOBEC cytidine deaminases such as APOBEC1, and their role in controlling mobile elements are discussed by Koito and Ikeda (2013).

The papers published in this Research Topic present findings and observations that are informative and insightful. They should serve not only as valuable references for the field, but provide future topics of research for investigators that should further our understanding of the retrovirus, retroelements and their restrictions.
